# Whispers beneath the soil: soybean-microbe communication pathways in the rhizosphere

**DOI:** 10.3389/fpls.2025.1686819

**Published:** 2025-10-22

**Authors:** Sau-Shan Cheng, Carolina A. Contador, Feng Zhang, Yun-Lam Ho, Hon-Ming Lam

**Affiliations:** ^1^ School of Life Sciences and Centre for Soybean Research of the State Key Laboratory of Agrobiotechnology, The Chinese University of Hong Kong, Hong Kong, Hong Kong SAR, China; ^2^ Institute of Environment, Energy and Sustainability, The Chinese University of Hong Kong, Hong Kong, Hong Kong SAR, China

**Keywords:** soybean, rhizospheric microbiota, sustainable agriculture, secondary metabolites, microbiota assembly, environmental stresses

## Abstract

The rhizosphere is a dynamic ecosystem that hosts diverse microbial communities, essential for nutrient cycling, and promoting plant health and resistance to environmental stresses and pathogens. Understanding the communication strategies between plant roots and these microbial communities is vital for sustainable agriculture, as these interactions can enhance crop resilience and productivity while reducing the need for chemical fertilizers. Extensive research has focused on how soybean plants shape the rhizosphere microbiota and the signaling processes that promote these interactions; however, many influencing factors, particularly environmental stresses, remain unexplored. Key elements, including soybean genetics, growth development stages, soil properties, agricultural practices, and environmental conditions, all play crucial roles in shaping microbial symbioses. This review examines the intricate interactions between soybean and their rhizospheric microbiota, emphasizing how various stresses affect these relationships. It also discusses the role of secondary metabolites from both microbes and soybean in facilitating communication, alongside other factors that significantly influence these microbial interactions and soybean productivity.

## 1 Introduction

The rhizosphere is a vibrant ecosystem filled with a diverse range of microorganisms, including bacteria, fungi, and protozoa (Philippot et al., 2013; Olanrewaju and Babalola, 2022). These microbial communities play critical roles in nutrient cycling, outcompeting pathogens, and helping plants cope with abiotic stress (Thepbandit and Athinuwat, 2024). Therefore, soil microbiota is crucial for maintaining soil fertility, health, and resilience in the face of an ever-changing environment.

Biodiversity within this niche is driven by chemical signals, particularly those metabolites present in plant root exudates, which attract specific microbial taxa and influence their activities in the rhizosphere. Root exudates contain a variety of signaling molecules, including phytohormones, flavonoids, organic acids, sugars, and amino acids. The species- and genotype-specific composition of these metabolites in turn affects the structure and function of the microbial community in the rhizosphere (Bukhat et al., 2020). At the same time, microbes produce quorum-sensing molecules, such as siderophores, volatile organic compounds and antibiotics, to facilitate their interactions with host plants and other microbes (Baloch et al., 2024). These interactions create a complex network of cross-talks that further refines the composition of the rhizospheric microbial community.

The rhizospheric microbiota of soybean (*Glycine max*), a major staple crop, has been extensively studied, particularly for its symbiotic relationship with rhizobia (Liu et al., 2018; Agyekum et al., 2025). These mutualistic bacteria form nodules in the roots of leguminous plants, thereby enhancing nutrient uptake by fixing atmospheric nitrogen into bioavailable forms. In addition to rhizobia, arbuscular mycorrhizal fungi (AMF) and plant growth-promoting rhizobacteria (PGPR) also contribute to supporting soybean growth and nutrient acquisition. Such beneficial interactions help alleviate biotic and abiotic stresses by modulating stress and defense responses in the host plant (Zaefarian and Rezvani, 2016; Porter et al., 2020).

The complex signaling network in the rhizosphere is influenced by plant species, genotypes, environmental stresses, and soil properties, which affect chemical signaling and the composition of microbial communities (Ramongolalaina, 2019; Han et al., 2023; Qu et al., 2023). Recent findings suggest that spatial heterogeneity and agricultural practices could impact the composition of the predominant species in soil microbial communities (Ren et al., 2025). Different soybean genotypes have been shown to assemble distinct rhizospheric microbial communities during plant development, from the seedling stage to mature seeds (Sohn et al., 2021; Qu et al., 2023). Additionally, the composition of key microbial taxa is correlated with nutrient cycling, soybean health, and yield (Ren et al., 2025).

Environmental stresses, such as drought, temperature extremes, and salinity, have been shown to influence the composition and quantity of root exudates (Moretti et al., 2021; McMillan et al., 2022; Han et al., 2023). These changes in exudates can alter the diversity and structure of rhizospheric communities, helping the plant improve its tolerance to stress. For instance, soybean treated with a bacterial consortium of *Bradyrhizobium* spp. and *Azospirillum brasilense*, along with the application of microbial secondary metabolites, demonstrated improved nodulation, growth development, grain yield, and increased tolerance to oxidate damage during dry spells (Moretti et al., 2020). Additionally, rhizobacteria that produce the extracellular enzyme 1-aminocyclopropane-1-carboxylate deaminase can mitigate the adverse effects of heat and water deficit by lowering ethylene concentration (McMillan et al., 2022).

Agricultural practices also have a significant influence in shaping microbial composition in the rhizosphere. Interspecific plant interactions can stimulate variations in root exudates, affecting the microbial community dynamics. For instance, intercropping soybean with various sugarcane cultivars has been shown to alter the diversity and composition of the rhizospheric bacterial community (Liu et al., 2021). Additionally, it has been demonstrated that nitrogen-fixing bacterial communities are enriched in soybean-maize intercropping systems, where such enrichment has been associated with increased community stability and enhanced resistance to pathogens (Chang et al., 2022; Dang et al., 2024).

**Figure 1:**
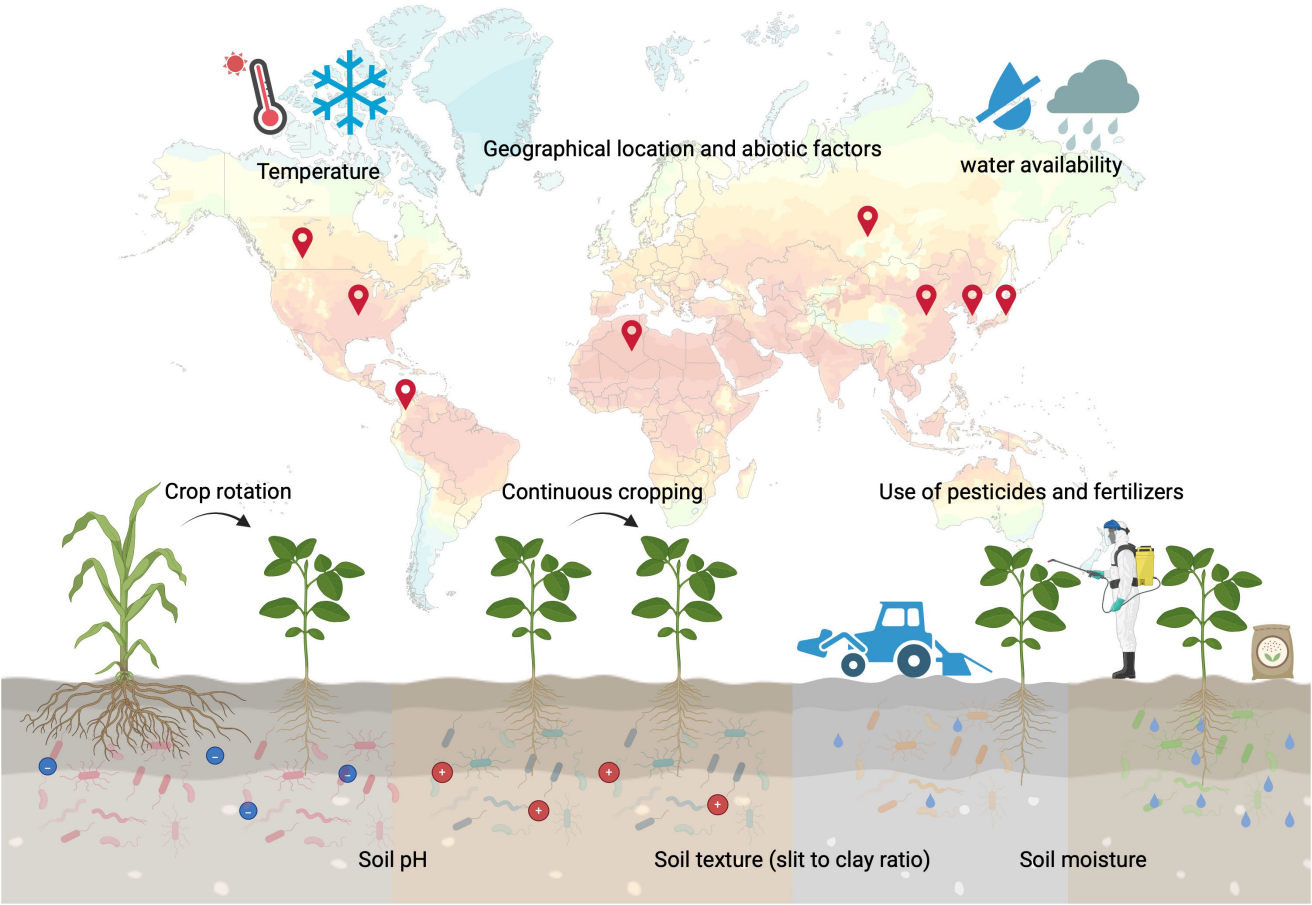
Overview of the abiotic and biotic stresses affecting microbial communities in the soybean rhizosphere. Interactions between plants, microbes and growth conditions shape the rhizosphere. Changes in plant growth and root exudate profiles influence the makeup of the rhizosphere microbial community, attracting specific microbial taxa that can adapt to the new conditions and requirements. This figure was created using BioRender (https://BioRender.com/p1ck2lu).

Moreover, the continuous cultivation of soybean also appears to alter the rhizobial community in the field. It has been reported that successive cropping of soybean resulted in a significant decrease in the proportion of *Bradyrhizobium diazoefficiens* USDA110 in the soil (Ramongolalaina, 2019). However, cultivars with high levels of root-secreted daidzein helped to maintain the USDA110 population in the rhizosphere despite continuous soybean cultivation (Ramongolalaina, 2019). Furthermore, different soil types exhibit varying rhizospheric communities that correlate with the presence of *Bradyrhizobium* and *Sinorhizobium* in the nodules (Han et al., 2020). An overview of the factors affecting rhizospheric microbial communities is summarized in **Figure 1**.

Understanding the signaling mechanisms that shape the rhizospheric microbiota is essential for promoting sustainable crop production and enhancing resilience to environmental stresses. In this review, we explored the secondary metabolites that mediate the two-way communication pathways between soybean and its rhizospheric microbiota, with a focus on how various abiotic stresses influence these interactions. We also examine additional factors that might influence these interactions.

## 2 Plant-to-microbe communication

Plants and soil microbes exude different metabolites and molecules to communicate with each other. Through their roots, plants secrete various metabolites, including flavonoids, hormones, and organic acids, which help recruit and repel microbes in the rhizosphere. These root exudates are modulated in response to factors such as nutrient requirements, environmental conditions, and plant development stages. For example, environmental stresses like drought or salt stress trigger the release of exudates, such as amino acids and organic acids, that attract microbial species adapted to thrive under such conditions and ameliorating stress (Yang et al., 2025). Some potential functions of these metabolites secreted by soybean for recruiting and interacting with microbes are discussed below.

### 2.1 Flavonoids

Flavonoids are a diverse group of naturally occurring polyphenolic compounds distributed across the plant kingdom (Brodowska, 2017; Ku et al., 2020). Soybean and other legumes are particularly rich in a distinctive subclass of flavonoids known as isoflavones. Isoflavones are structurally defined by a 3-phenylchromen skeleton, where the B ring is attached to the C-ring at position 3, while flavones share a B ring at position 2 (Brodowska, 2017; Ku et al., 2020).

Owing to the presence of the phenolic hydroxyl group on the nucleus of flavonoid molecules, flavonoids constitute a secondary ROS-scavenging system to balance the oxidative levels in plants or, upon consumption, in herbivores and omnivores including humans (Fini et al., 2011; Ku et al., 2020). Other than serving as a ROS-scavenger, flavonoids, including isoflavones, could also serve as signaling molecules and allelochemicals for attracting or repelling microbes to the soybean rhizosphere, helping to shape the composition of the microbial community (Kosslak et al., 1987; Banfalvi et al., 1988; Morris and Ward, 1992; Smit et al., 1992; Graham et al., 2007; Dardanelli et al., 2010; Guo et al., 2011; White et al., 2017; Okutani et al., 2020).

Soybean isoflavones promote plant interactions with rhizobia. Isoflavones such as genistein and daidzein have been shown to induce the expression of nodulation-related genes in rhizobia (Kosslak et al., 1987; Banfalvi et al., 1988; Smit et al., 1992). Coumestrol, one of the abundant coumestan isoflavones in the soybean leaf (Yuk et al., 2011), was also shown to induce the nodulation (*nod*) genes in rhizobia and enhance soybean nodulation by increasing the number of nodules (Lee et al., 2012). Isoflavone synthase (IFS) is a key enzyme for isoflavone biosynthesis; the *IFS-*RNAi soybean mutant showed a significant reduction in root isoflavonoids at the rhizosphere (Graham et al., 2007). Rhizospheric microbial composition analyses of the *IFS-*RNAi soybean mutant revealed alterations in the bacterial taxa in the proximal soil, suggesting that isoflavonoids might inhibit the presence of *Comamonas* spp. while promoting *Xanthomonas* spp. (White et al., 2017). Similarly, soil daidzein and genistein concentrations secreted by cultivated soybean plants showed a positive correlation with the relative abundance of soil fungi species (Guo et al., 2011). Meanwhile, genistein has been shown to exhibit a positive correlation with the overall abundance Gram-positive and aerobic bacteria (Guo et al., 2011). Increased concentrations of daidzein in soil were associated with an increased abundance of *Comamonadaceae* (Okutani et al., 2020).

However, the flavonoids do not only facilitate beneficial plant-microbe interactions. Genistein and daidzein cannot only draw in the symbiont, but also the soybean pathogen *Phytophthora sojae*, through chemotactic attraction (Morris and Ward, 1992). *P. sojae* zoospores showed chemotaxis towards genistein and daidzein, which induced rapid encystment and germination of the fungus upon contact (Morris and Ward, 1992). Moreover, the study using the *IFS*-RNAi soybean mutant suggested that isoflavones were involved in programmed cell death in roots that contributes to the race-specific resistance to *P. sojae* (Graham et al., 2007). The accumulation of daidzein and genistein in consecutive soybean monocultures resulted in a shift in the soil microbial community, with an enrichment of *Fusarium* spp. in the soybean rhizosphere (Wang et al., 2020).

The exudation of flavonoids in soybean root exudates is sensitive to environmental stress (Kumar et al., 2023; Yao et al., 2024), potentially influencing the interaction with microbes. In soybean nodules, it has been reported that the distribution of isoflavonoids, malonyldaidzin and malonylgenistin, showed a shift from the cortex to the base of the nodules, which are closer to the root, under drought or alkaline stress conditions (Zhang et al., 2025). The spatial redistribution of signaling molecules in response to stress might correlate with enhanced soybean-rhizobia symbiosis, strengthening adaptability under adverse conditions (Zhang et al., 2024, 2025). In another study, dual inoculation with rhizobia and arbuscular mycorrhizal fungi has been found to improve soybean tolerance to drought stress, resulting in better seed yield and quality (Igiehon et al., 2021).

### 2.2 Soyasaponins

Soyasaponins are oleanane triterpenoid glycosides that are commonly produced by leguminous plants (Zhang and Popovich, 2009). Soyasaponins can be categorized into two major groups based on their aglycones: Group A and Group B (Zhang and Popovich, 2009). Meanwhile, depending on whether they are conjugated with a 2,3-dihydro-2,5-dihydroxy-6-methyl-4-pyrone (DDMP) or a ketone at carbon 22, DDMP soyasaponins and group E soyasaponins, respectively, can be derived from group B soyasaponins (Zhang and Popovich, 2009; Guang et al., 2014). Although the biological function of soyasaponins in the soil is not fully understood, it has been demonstrated that soybean selectively secretes different groups of soyasaponins at different developmental stages, and such secretion patterns have been proposed to influence the microbial composition and the activities of both macroorganisms and microorganisms in the soil, based on the relative concentrations of each type of soyasaponin (Tsuno et al., 2018). It has been demonstrated that soyasaponin Bb, one of the most abundant forms of soy saponin secreted in the soil, was potentially utilized by the bacterial community (Fujimatsu et al., 2020). Moreover, specific bacterial families, such as *Novosphingobium* spp. and *Sphingomonadaceae*, exhibited a dosage-dependent enrichment by soyasaponin Bb, suggesting a potential role for soyasaponins in fostering communications between soybean and soil microorganisms (Fujimatsu et al., 2020).

### 2.3 Phytohormones

Phytohormones are signaling molecules that regulate soybean growth, development, and stress responses (Miransari, 2016). Similar to other plants, the disequilibrium in the phytohormone levels was observed in soybean upon abiotic and biotic stress (Ku et al., 2018; Beyer et al., 2021; Shaffique et al., 2023). In addition, research has highlighted their importance in influencing soybean interactions with soil microbes.

Strigolactones (SLs) are a group of carotenoid-derived terpenoid lactones that have a butenolide ring (D ring) in common (Al-Babili and Bouwmeester, 2015). Exogenous supplementation of strigolactones in soybean seedlings showed enhanced tolerance to alkaline stress (Chen et al., 2022). Additionally, foliar application of strigolactones has been shown to improve osmoregulation and antioxidant defense under drought stress (Cao et al., 2024).

Soybean mutants with the SL biosynthesis and signaling genes, *GmMAX3*, *GmMAX1a*, and *GmMAX4a*, knocked down or overexpressed showed altered expression of genes involved (Haq et al., 2017; Rehman et al., 2018). *GmMAX1a-* and *GmMAX4a*-knockdown soybean lines were reported to have a defective SL biosynthesis pathway, resulting in reduced root hair length and suppressed nodulation (Rehman et al., 2018). Knocking down *GmD53a*, a proposed SL suppressor, on the other hand, resulted in an increased number of nodules in the soybean root (Rehman et al., 2022). A soybean mutant overexpressing the strigolactone biosynthesis gene, *GmMAX1d*, had a highly divergent rhizospheric bacterial composition with the specific enrichment of the bacterial genera, *Shinella* and *Bdellovibrio* (Liu et al., 2020). Moreover, the *GmMAX1d-*overexpressing lines also led to the specific enrichment of the fungal species, *Fusarium solani* (Liu et al., 2020).

Auxins are a key group of growth hormones responsible for regulating plant development, germination and root architecture, whilst the indole-3-acetic acid (IAA) is the most abundant form of auxins in plants (Spaepen et al., 2007; Gomes and Scortecci, 2021). Auxins share a similar chemical structure composed of a pyrrole ring connected to a benzene ring and a carboxyl group (Gomes and Scortecci, 2021). The endogenous level of auxin might also affect how plant adapted to stress. For instance, heat-stress-tolerant soybeans are often characterized with high levels of IAA and GAs (Park et al., 2017). Auxins are also involved in regulating the interactions between microbes and soybean. It has been reported that the moderately susceptible soybean cultivar Sloan has a higher level of the auxin catabolite, IAA-Ala, than the moderately resistant soybean cultivar Conrad upon challenge by *Phytophthora sojae* (Stasko et al., 2020). Furthermore, the cultivar Conrad showed a higher expression of the *Pin-formed* (*GmPIN*) auxin efflux transporter gene, suggesting its capability for IAA catabolism in the resistance against pathogens (Stasko et al., 2020). The application of auxins also helped control the spread of antibiotic-resistant bacteria in soybean seedlings (Li et al., 2025).

Ethylene (ET), the simplest alkene characterized by the presence of carbon-carbon double bonds, exerts a dual function in regulating both growth and senescence, depending on its concentration and timing of application (Iqbal et al., 2017). Soybean treated with the ET inducer, ethephon, activates the ET biosynthesis genes and results in improved resistance against *Fusarium virguliformes* by inhibiting the fungal growth (Abdelsamad et al., 2019). An ET-insensitive mutant with mutated ethylene receptors, GmEtr1 and GmEtr2, showed a lowered sensitivity and disease severity upon infection with *Pseudomonas syringae* pv. *glycinea* and *Phytophthora sojae*; meanwhile developed a more severe symptom towards *Septoria glycines* and *Rhizoctonia solani* infection (Hoffman et al., 1999). The ET level in wild-type soybean was induced upon inoculation with *Bradyrhizobium japonicum* (Suganuma et al., 1995). In contrast, such an induction in the ET level was not observed in the non-nodulating soybean mutant when inoculated with the heterologous rhizobium, *Rhizobium leguminosarum* bv. *Viciae* (Suganuma et al., 1995). The overexpression of ET-responsive transcription factors*, GmENS1* and *GmENS2*, accelerated senescence in soybean nodules; meanwhile, knocking down of these two transcription factors resulted in delayed senescence (Xiao et al., 2024). The supplementation of the ethylene precursor, 1-aminocyclopropane-1-carboxylate (ACC), in soybean has been shown to improve tolerance to alkaline and drought stresses by increasing the secretion of flavonoids into the rhizosphere (Hasan et al., 2024). Moreover, DNA-based qPCR revealed a significant increase in both 16S bacterial abundance and ITS fungal abundance with soils supplemented with ACC under combined alkaline and drought conditions (Hasan et al., 2024). This highlights the potential of phytohormones and their precursors in modulating soybean gene expression, recruiting diverse microbial communities for stress amelioration (Hasan et al., 2024).

Salicylic acid (SA) is a monohydroxybenzoic acid that is present in soybean root exudate and plays a crucial role in plant innate immunity and resistance to pathogens (Spoel and Dong, 2024; Yao et al., 2024). SA levels are positively correlated to the induced resistance in plants against many microbes. SA is essential for soybeans to defend against soybean mosaic virus (SMV) (Shang et al., 2023). The induced expression of the *SA carboxyl methyltransferase* gene, *GmSAMT1*, conferred better resistance against the soybean cyst nematode (Lin et al., 2013). Similarly, exogenous SA application leads to induced resistance against *Fusarium solani* (Bawa et al., 2019), while exogenous SA treatment inhibits the early formation of nodules (Sato et al., 2002). Although the impact of SA on the microbial community in the soybean rhizosphere is not well understood, in Arabidopsis, mutants with disrupted SA biosynthesis or signaling resulted in the alteration of root microbiome composition compared to the wild-type plants (Lebeis et al., 2015).

### 2.4 Organic acids and other metabolites

Plant root exudates often contain various metabolites, including sugars, amino acids, organic acids, and peptides, that contribute to shaping the rhizospheric microbiome (Sugiyama, 2019; Ma et al., 2022; Ku et al., 2025). The soybean root exudate contains *N*-cyclohexylformamide, xanthine, and 2,4,5-trimethoxybenzoic acid, which help recruit *Pseudomonas* spp. (Zheng et al., 2024). Glutamate, aspartate, and dicarboxylic acids from the soybean root exudate serve as natural chemoattractants for rhizobia (Barbour et al., 1991), facilitating the formation of root nodules. Amino acids, including alanine, asparagine, glutamine, serine, and threonine, in the soybean root exudate induce the chemotaxis of a potential biocontrol agent, *Bacillus megaterium* strain B153-2-2, towards the soybean host to help fight against Rhizoctonia root rot (Zheng and Sinclair, 1996). The overexpression of a soybean predicted amino acid transporter, *Rhg1‐GmAAT*, enhances glutamate transport and activates the jasmonic acid (JA) pathway, which increases resistance to the soybean cyst nematode (Guo et al., 2019). Furthermore, certain amino acids, including proline and glutamate, have been found to provide protective effects for crops under various stress conditions (Teixeira et al., 2020; Saleem et al., 2024).

In addition, it has been proposed that citric acid in the soybean root exudate acts as a repellent against *Phytophthora sojae* zoospores (Zhang et al., 2019). Salt-stressed soybean plants secrete purines and xanthine, which may induce chemotactic responses in *Pseudomonas* spp., with confirmed salt tolerance enhancing capability of wild soybean (Zheng et al., 2024). Moreover, exogenous application of xanthine to non-stressed soybean resulted in shifts in the microbiota community, mirroring the microbial composition observed in the rhizosphere of salt-stressed soybean (Zheng et al., 2024).

## 3 Microbe-to-plant signaling

Microbes can also secrete proteins or peptide effectors to enhance infection or promote symbiosis with host plants. These microbes may secrete enzymes or phytohormones to modulate the phytohormones in the rhizosphere or *in planta*, thereby facilitating infection or promoting plant growth under various conditions. Some microbes can also secrete metabolites to modulate the plant gene expression or modify the plant root architecture to facilitate the interaction with soybeans. Below, some potential functions of these metabolites secreted by microbes to support their interactions with soybean are discussed.

### 3.1 Proteins and peptides

Pathogenic microbes often secrete various effectors to facilitate infection, and effector recognition significantly influences the virulence of these microbes in plants including soybean (Ku et al., 2020). Meanwhile, rhizobia and AMF often produce specialized effectors to promote beneficial symbiosis with soybean (Maillet et al., 2011; Lerouge et al., 1990; Schaarschmidt et al., 2013; Dénarié et al., 1992). Upon recognizing the nodulation inducer released from the legume host, rhizobia produce Nod factors, which are crucial for the formation of nodules and the maintenance of their symbiotic association with the legume (Lerouge et al., 1990; D’Haeze and Holsters, 2002). NodD proteins, activated by plant or environmental signals, induce the transcription of structural *nod* operons, leading to the production of Nod factors (Dénarié et al., 1992). Multiple copies of *nodD* might be present in the rhizobium to respond to various inducers (Dénarié et al., 1992; Van Rhijn et al., 1994). Nod factors consist of an acetyl glucosamine oligomeric backbone linked with various long-chain fatty acyl groups and functional groups, also known as lipo-chitooligosaccharides (LCOs) (Dénarié et al., 1996). Different forms and structures of Nod factors contribute to host specificity in rhizobium-plant interactions (Lerouge et al., 1990; Dénarié et al., 1992). The soybean plant recognizes these Nod factors through a lipo-oligochitin LysM-type receptor kinase (Indrasumunar et al., 2011). The recognition of the Nod factors leads to the reprogramming of the root system, resulting in the curling of root hairs and the development of infection threads, which facilitate the entry of rhizobia and the formation of nodules (Gresshoff, 2003). Similarly, fungi might release Myc factors, which are composed of a mixture of chitin oligomers (COs) and LCOs that serve as symbiotic signals for stimulating arbuscular mycorrhiza formation in legume roots (Maillet et al., 2011). However, the receptor kinase for Myc factor recognition in soybean remains unexplored (Schaarschmidt et al., 2013).

Many effectors are proteins or peptides. One of the best-studied conserved epitopes, flg22, is a 22-amino-acid peptide present in the bacterial protein, flagellin, and is perceived by the leucine-rich repeat-containing receptor-like kinase (LRR-RLK), FLAGELLIN SENSING2 (Boutrot and Zipfel, 2017). The ability to evade pathogen-associated molecular pattern (PAMP) recognition by the host contributes to infection and pathogen proliferation (Pfund et al., 2004; Chen et al., 2023). For instance, soybean GmFLS2, which contains an exceptional flg22-binding domain different from other legumes, allows the perception of the polymorphic flg22 from *Ralstonia solanacearum*, contributing to the soybean resistance against *Ralstonia* (Chen et al., 2023). Conversely, silencing *GmFLS2* reduces the resistance of soybean plants to *Pseudomonas syringae* pv. *glycinea* by compromising the GmMAPK signaling pathway through a deficiency in Psg-flg22 recognition (Tian et al., 2020). The N-terminal domain, Arg-X-Leu-Arg (RXLR), in the *Phytophthora sojae* effector, PsAvh238, helps the pathogen evade host recognition with just one mutation in the nucleotide sequence while retaining the ability to suppress plant immunity and enhance infection (Yang and Guo, 2018).

Some microbial proteins modulate the levels of phytohormones as a strategy for promoting interactions. For example, the *Phytophthora* effector PsAvh238 targets the ET biosynthesis enzyme 1-aminocyclopropane-1-carboxylate synthase (ACS) to destabilize Type 2 GmACSs and downregulate ET biosynthesis to facilitate host infection (Yang et al., 2019).

**Figure 2:**
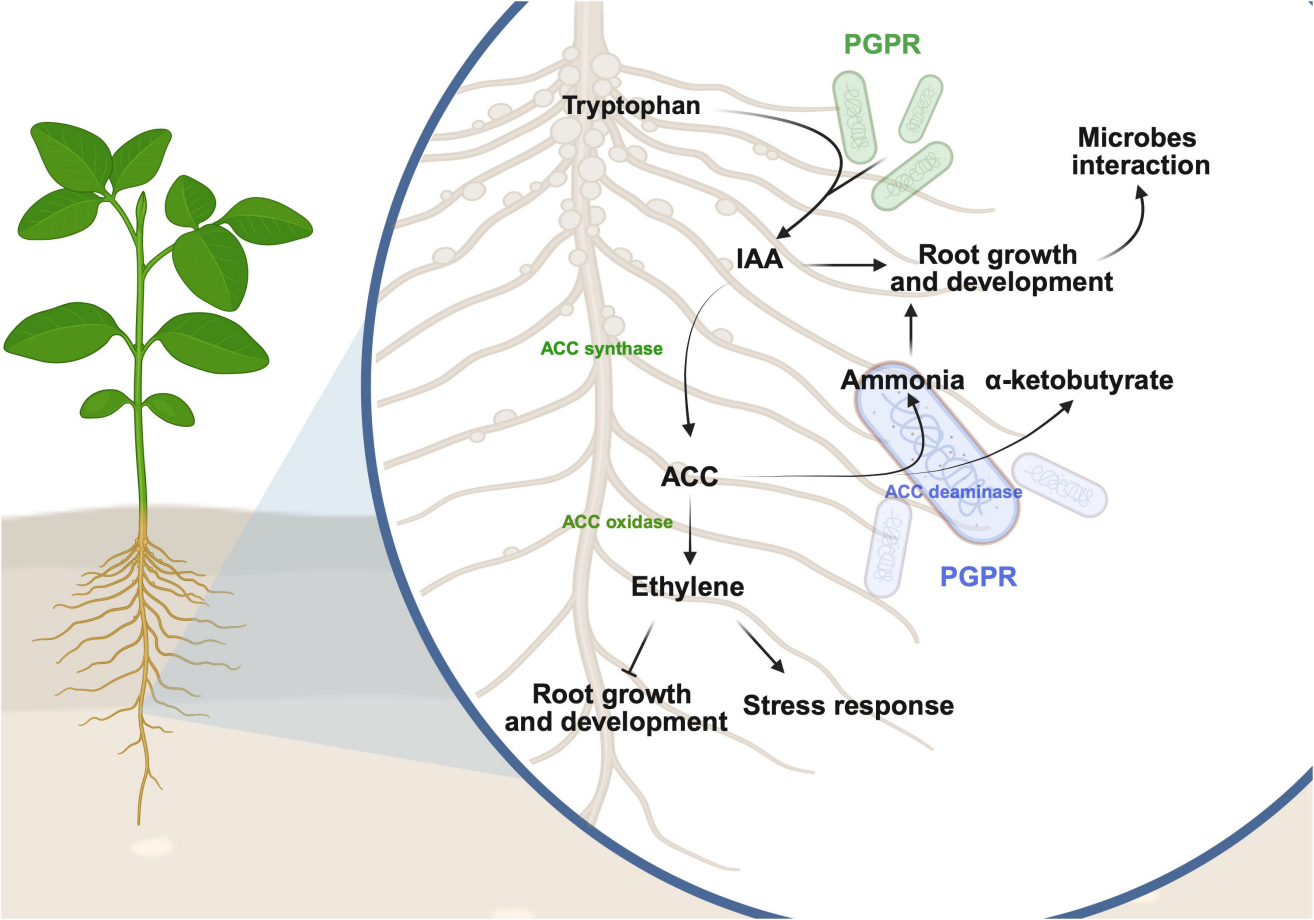
Proposed mechanisms of how plant growth-promoting rhizobacteria (PGPR) could influence plant-microbe interactions. Some IAA-producing PGPR can convert tryptophan from soybean into indole-3-acetic acid (IAA), a hormone that promotes root growth. IAA can then be converted to 1-aminocyclopropane-1-carboxylic acid (ACC) by ACC synthase and further into ethylene by ACC oxidase in soybean. Ethylene is a phytohormone that inhibits root growth and development while enhancing plant stress responses. Some PGPR possess ACC deaminase activity that converts ACC to ammonia and α-ketobutyrate. The ammonia produced could be used as an essential macronutrient for plant growth. It is proposed that the IAA and ammonia produced from the beneficial interactions between soybean and microbes can foster the growth and development of soybean roots, ultimately enhancing the total surface area for interacting with the soil microbiota. This figure was created using BioRender (https://BioRender.com/u4p3b0o).

Ethylene regulates plant growth, development, and defense against stresses, however, the excessive accumulation of ethylene and its precursor ACC, often leads to the inhibition of plant growth under various environmental stresses such as heat, salinity, drought, flooding and the low bioavailability of nutrients (Tao et al., 2015; Nazir et al., 2024). Some soybean-interacting microbes possess ACC deaminase activities (Khan et al., 2019; Dubey et al., 2021; Roriz et al., 2023; Win et al., 2023) that can lower the ET accumulated in plants by deaminating ACC into α-ketobutyrate and ammonia (NH_3_) (Khan et al., 2019; Shahid et al., 2023). Lower ET levels coupled with increased levels of NH_3_, is often associated with improved root growth, which might eventually promote plant-microbe interactions (Dubey et al., 2021; Huang et al., 2022). The potential functions of the microbial metabolites in facilitating the interactions between soybean and microbes is illustrated in **Figure 2**.

Certain soybean-interacting beneficial microbes produce enzymes that convert nutrients from insoluble forms into more bioavailable forms in the soil. This process enhances nutrient availability, promotes root growth, and indirectly fosters plant-microbe interactions by expanding the areas available for these interactions (Massucato et al., 2025). Both phosphatases and phytase are essential enzymes that mineralize phosphate compounds, releasing orthophosphate into the soil in a bioavailable form for soybean uptake (Rawat et al., 2021). The bacterial genus *Cupriavidus* has been shown to exhibit acid phosphatase activity and is particularly enriched in the rhizosphere of the soybeans cultivated under phosphate-deficient acidic soil (Chen et al., 2024). Moreover, commercial inoculants, including *Priestia megaterium* (Ag87), *Lysinibacillus* sp. (Ag94), *Priestia megaterium* (B119), and *Bacillus subtilis* (B2084), exhibited phytase and both acid and alkaline phosphatase activities, which help improve phosphorus use efficiency and dependency in soybean cultivation under low-phosphorus soils (Massucato et al., 2025).

### 3.2 Phytohormones

Exogenous application of phytohormones via root or foliar application might help ameliorate the negative effects of abiotic stresses on soybean plants (Noor et al., 2022; Shaffique et al., 2023). Many of the soybean-interacting microbes produce phytohormones that might lead to better survival of the plant (Egamberdieva et al., 2017; Hamayun et al., 2017; Park et al., 2017; Kang et al., 2019; Khan et al., 2019, 2020; Dubey et al., 2021). For instance, the soybean growth-promoting bacterium *Bacillus aryabhattai* strain SRB02 could produce abscisic acid (ABA), IAA, cytokinin, and gibberellic acid (GA), which in turn modulate the endogenous levels of these soybean phytohormones when under oxidative and nitrosative stress. An endophytic fungus, *Curtobacterium oceanosedimentum* SAK1, produces ABA, IAA, and GA that enhance the development of root architecture under salt stress (Khan et al., 2019). Endophytic bacteria *Bacillus cereus SA1* could improve soybean heat tolerance while producing biologically active gibberellin and IAA (Khan et al., 2020). *Bacillus tequilensis* SSB07, a soybean growth-promoting bacterium under heat stress, secretes GA, IAA and ABA (Kang et al., 2019). Several IAA-producing bacterial isolates, AKAD A1-1, AKAD A1-2, and AKAD A1-16, that closely resemble *Bacillus cereus* (MN079048), *Pseudomonas otitidis* (MW301101), and *Pseudomonas* sp. (MN079074), promote soybean growth under drought stress (Dubey et al., 2021). *Pseudomonas putida* TSAU1 forms a tripartite interaction with rhizobium, *Bradyrhizobium japonicum* USDA 110 and soybean seed cultivar YC03-3, producing IAA while alleviating soybean salt stress (Egamberdieva et al., 2017).

Some studies have found that phytohormones produced by the microbes might help to modulate the interactions between microbes and the plant. A *Rhizobium japonicum* mutant B-14075 that catabolizes tryptophan to IAA could enhance nodule formation in soybean (Kaneshiro and Kwolek, 1985). Soybean inoculated with B-14075 showed a significant increase in root weight and nodule volume, a result of the enhanced nodulation (Kaneshiro and Kwolek, 1985). It was proposed that root-accumulated IAA or auxin promoted the reallocation of nutrients to the root, thereby improving nutrient acquisition by the microbe (Altman and Wareing, 1975; Kaneshiro and Kwolek, 1985).

The soybean pathogens, *Xanthomonas campestris* pv. *glycines* and *Pseudomonas syringae* pv. *syringae*, are capable of producing various indoles including IAA (Fett et al., 1987). IAA was shown to have multiple effects on plant tissues, which might promote bacterial multiplication (Fett et al., 1987). A study on *Arabidopsis thaliana* showed that auxin signaling is required for susceptibility to *Pseudomonas syringae* DC3000 (PtoDC3000). The disruption of auxin signaling in plants diminished the growth of the IAA-producing pathogen PtoDC3000, and therefore reduced the susceptibility of the plants (Djami-Tchatchou et al., 2020). In contrast, elevated endogenous IAA levels in plants are associated with the suppression of the SA-mediated defense, promoting PtoDC3000 pathogenesis (Djami-Tchatchou et al., 2020). Moreover, it was proposed that exogenous auxin could stimulate the rapid elongation of plant tissues by increasing cell wall extensibility, thereby facilitating the interactions between microbes and the plant host (Cosgrove, 1993; Kunkel and Harper, 2018).

### 3.3 Organic acids and other metabolites

Although their mode of recruitment and interaction mechanism for the plant growth-promoting bacteria remains largely unexplored, the ability of microbes that are capable of producing growth-promoting or stress-relieving organic acids and other secondary metabolites is often enriched in the soybean rhizosphere. For instance, phosphate-solubilizing bacteria Ag94 and Ag87 secrete lactic, malic, and acetic acids in the presence of insoluble phosphate (Massucato et al., 2025). Meanwhile, B119 and B2084 produce gluconic, malic, and acetic acids (Massucato et al., 2025). These organic acids help acidify the rhizosphere, chelate cations, and promote the release of soluble orthophosphate in soil, thereby improving phosphate use efficiency in soybeans (Lei et al., 2025). A plant growth-promoting PGPR *Bacillus aryabhattai* SRB02, isolated from a soybean field, secretes low concentrations of butanoic acid. This secretion promotes soybean growth in both shoots and roots, increase chlorophyll content, and boosts both fresh and dry weight (Mun et al., 2024). A study on mutants of *P. putida* KT2440, mus-20, mus-42, and EU206 strains has shown elevated production of exopolysaccharides (EPS) (Martínez-Gil et al., 2013). Inoculation of these mutant strains to soybean demonstrated improved germination and growth parameters under saline conditions (Costa-Gutierrez et al., 2020). Other studies utilizing other crops have indicated that the EPS produced by the plant growth-promoting bacteria might contribute to the enhanced water availability in soil while promoting the binding of cations, including sodium ions (Alami et al., 2000; Upadhyay et al., 2011).

**Table 1** summarizes the metabolites involved in soybean-microbe interactions.

**Table 1:** Examples of metabolites involved in soybean-microbe interactions and their known functions.

Source	Metabolites	Microbes	Soybean cultivar	Functions	References
Soybean	Coumestrol	*Bradyrhizobium japonicum* USDA110	Specific cultivar not mentioned	• Induced expression of *nodD1* and *nodABC* • Induced biofilm formation of *Bradyrhizobium*	(Lee et al., 2012)
	Daidzein and genistein	*Phytophthora sojae*	*In vitro* study	Hastened the encystment and germination of zoospores	(Morris and Ward, 1992)
	5-Deoxyisoflavonoids	*Phytophthora sojae*	*Glycine max* cv. Williams 79 *Glycine max* cv. Williams 82	Triggered a cell death response in roots upon contact with cell wall glucan elicitor of *P. sojae*.	(Graham et al., 2007)
	Strigolactones (SL)	*Bradyrhizobium japonicum* strain USDA110	*GmMAX3b* overexpressor and knockdown mutant in *Glycine max* cv. “Tianlong No.1” as the genetic background	• *GmMAX3b* overexpressor with a higher level of SL showed increased nodule number, and *GmMAX3b* knockdown mutant with reduced nodule number • Reduced root hair length	(Rehman et al., 2018)
	Strigolactones (SL)	*Bradyrhizobium japonicum*	*Gm53a* knockdown mutant in *Glycine max* cv. “Tianlong No.1” as the genetic background	Knockdown of *Gm53a*, an SL suppressor, resulted in an increased number of nodules	(Rehman et al., 2022)
	Ethylene (ET)	*Bradyrhizobium japonicum*	*Glycine max* (L.) Merr. cv. Hakuchou	Induction in the ET level for successful nodulation	(Suganuma et al., 1995)
	Ethylene (ET)	*Bradyrhizobium diazoefficiens* strain USDA 110	*Glycine max* cv. Williams 82	Increased ET level promoted soybean nodule senescence	(Xiao et al., 2024)
	*N*-cyclohexylformamide, xanthine, and 2,4,5-tri- methoxybenzoic acid	*Pseudomonas*	*Glycine soja*	Recruited root-associated *Pseudomonas* for better salt tolerance	(Zheng et al., 2024)
	Glutamate, aspartate, and dicarboxylic acids	*Bradyrhizobium japonicum* strain USDA110	*Glycine max* cv. Essex	As a natural chemoattractant of *B.japonicum* strain USDA110	(Barbour et al., 1991)
	Amino acids such as alanine, asparagine, glutamine, serine, and threonine	*Bacillus megateriumstrain* B153-2-2	*Glycine max* cv. Hack	As a natural chemoattractant of *B.megateriumstrain* B153-2-2, and used as a potential biocontrol agent against Rhizoctonia root rot	(Zheng and Sinclair, 1996)
	Citric acid	*Phytophthora sojae*	*Glycine max* cv. Yidianhong, *Glycine max* cv. Williams 82	As a repellent of *P. sojae*	(Zhang et al., 2019)
Exogenous application	Salicylic acid (SA)	*Fusarium solani*	*Glycine max* cv. Jiuyuehuang	Increased SA level confers better resistance of soybean seedlings against *Fusarium solani* infection	(Bawa et al., 2019)
	Salicylic acid (SA)	*Bradyrhizobium japonicum* strain USDA110	*Glycine max* cv. Williams 82	Reduced nodules size and numbers	(Sato et al., 2002)
Microbes	Nod factors	*Rhizobia*	Not Applicable	• Recognition of the Nod factors by soybean led to the reprogramming of the root system • Induced the curling of root hairs and the development of infection threads for the formation of nodules	(Dénarié et al., 1992; Van Rhijn et al., 1994; Gresshoff, 2003)
	Myc factors (chitin oligomer and lipo-chitooligosaccharides)	Arbuscular mycorrhiza fungi (AMF)	Not Applicable	As a symbiotic signal for stimulating arbuscular mycorrhiza formation in legumes	(Maillet et al., 2011)
	flg22	*Ralstonia solanacearum*	Specific cultivar not mentioned	• PAMP recognition led to resistance against pathogen. • The evasion of PAMP recognition led to infection and pathogen proliferation	(Chen et al., 2023)
	PsAvh238	*Phytophthora*	Specific cultivar not mentioned	Destabilized Type 2 GmACSs and suppressed Type 2 ACS-catalyzed ET biosynthesis to facilitate infection	(Yang and Guo, 2018)
	ACC deaminase	ACC deaminase-producing bacteria	Not Applicable	• Reduced the salt-induced ET production and improved nutrient uptake of soybean • Reduced ET level often promoted root growth for better interaction with microbes	(Dubey et al., 2021; Huang et al., 2022; Win et al., 2023)
	Indole-3-acetic acid (IAA)	*Rhizobium japonicum* mutant B-14075	*Glycine max* cv. Clark L-1	• Increase in root weight and nodule volume • Enhanced the reallocation of nutrients to the root which improved nutrient acquisition by the microbe	(Altman and Wareing, 1975; Kaneshiro and Kwolek, 1985)
	Indole-3-acetic acid (IAA)	*Xanthomonas campestris* pv. *glycines* and *Pseudomonas syringae* pv. *syringae*	*In vitro* study	Promoted bacterial multiplication	(Fett et al., 1987)
	Phosphatases, phytase and organic acids, such as lactic, malic, acetic, and gluconic acids	*Priestia megaterium* (Ag87), *Lysinibacillus* sp (Ag94), *Priestia megaterium* (B119) and *Bacillus subtilis* (B2084)	*In vitro* study and field study using *Glycine max* cv. Credenz Result I2X	Solubilize phosphorus for better soybean uptake and improved yield	(Massucato et al., 2025)
	Butanoic acid	*Bacillus aryabhattai* SRB02	*Glycine max* cv. Daewon	Promote soybean growth in both shoots and roots, chlorophyll content, fresh weight, and dry weight	(Mun et al., 2024)
	Exopolysaccharides	*P. putida* KT2440, strain mus-20, mus-42, and EU206	*Glycine max* variety A8000	Improved germination and alleviated salt stress phenotype of soybean	(Costa-Gutierrez et al., 2020)

PAMP, pathogen-associated molecular pattern.

## 4 Environmental factors and soil conditions affecting soybean-microbe signaling and interactions

The diversity of the microbial community could be influenced by the physicochemical properties of the soil, thus affecting plant-microbe interactions within the soil. Studies have suggested that the compositions of bacterial and fungal communities are correlated with specific soil properties such as the silt-to-clay ratio, pH, and carbon-to-nitrogen (C:N) ratio (Fierer and Jackson, 2006; Lauber et al., 2008; Labouyrie et al., 2023). Bacterial biodiversity was generally highest in neutral soil, while it decreases with lower pH (Fierer and Jackson, 2006). Moreover, the types of dominant microbes might also be affected by soil properties. For instance, *Acidobacteria* spp. were enriched in soil with a higher pH, while *α-Proteobacteria* were depleted (Lauber et al., 2008). Similarly, the fungal abundance of *Agaricales* spp. was positively associated with C:N, whereas that of *Sordariomycetes* was negatively correlated (Lauber et al., 2008).

Various soil physicochemical properties, including pH, temperature, water content, salinity, and oxygen availability, can significantly influence the interactions between soybean and rhizobia. In addition, the compatibility of rhizobia with existing microbiota in the soil also plays a curial role in nodulation and nitrogen-fixing efficiency (Ji et al., 2017; Han et al., 2020; Zhang et al., 2024). Two major soybean-interacting rhizobial genera, *Bradyrhizobium* and *Sinorhizobium* (*Ensifer*), show disparate competitiveness and nodulation abilities under different soil conditions. Research suggested that *Bradyrhizobium* is the dominant genus in neutral to acidic soils, while *Sinorhizobium* prevails in alkaline–saline soils (Man et al., 2008; Han et al., 2009; Li et al., 2011). Despite the adaptability of rhizobia to different soil conditions, the composition of the soil microbiota could also influence the growth and nodulation of rhizobia (Han et al., 2020). For example, *Bacillus cereus* promotes the growth and nodulation of *Sinorhizobium* spp. while suppressing *Bradyrhizobium* spp. (Han et al., 2020). Furthermore, the interactions between *B. cereus and Sinorhizobium* spp. alleviated the negative impacts of saline-alkaline conditions on nodulation (Han et al., 2020). The amount of active water-soluble humic acid, a key carbon source in the soil, has a positive effect on nodulation by suppressing soybean immunity and ET production (Li et al., 2024). Also, a higher concentration of potassium humate, a humic product that includes both humic and fulvic acids, is associated with an increased number of nodules when soybean plants were inoculated with *Bradyrhizobium* spp. (Reis De Andrade Da Silva et al., 2021; Canellas et al., 2023).

Distinct land-use types do not always support unique soil fungal or bacterial communities, but they are more likely correlated with altered relative abundances of these communities (Fierer and Jackson, 2006; Lauber et al., 2008; Yuan et al., 2021; Labouyrie et al., 2023). Certain land-use management practices, such as monoculture and intercropping, may lead to shifts in the microbial community (Zhu et al., 2013; Hamid et al., 2017; Chang et al., 2020; Wang et al., 2020). For instance, soybean monoculture increases the population of soybean cyst nematode (SCN), *Heterodera glycines*, in the first five consecutive years of cultivation (Zhu et al., 2013). Surprisingly, under a prolonged period of monoculture, the SCN population was actually repressed, accompanied by an increased abundance of *Streptomyces* and *Rhizobium* spp. (Zhu et al., 2013). Similarly, the long-term practice of soybean monoculture alters microbial communities, leading to increased levels of *Pseudomonas*, *Purpureocillium*, and *Pochonia* spp. that help suppress SCN (Hamid et al., 2017). Continuous soybean monoculture also resulted in the enrichment of the *Fusarium* community in the soybean rhizosphere (Wang et al., 2020). Meanwhile, another study found that continuous soybean monoculture enriched *Fusarium* populations with higher pathogenicity than the soybean-maize intercropping system, contributing to an increased severity and occurrence of soybean root rot (Chang et al., 2020).

A study on the soybean-maize intercropping system demonstrated the differential enrichment of amino acids and organic acids in plant root exudates compared to the respective monoculture systems (Zhang et al., 2024). The application of the root exudate from a soybean-maize intercropping system to soybean monoculture showed an increase in the relative abundance of the AMF, *Glomus Glomeraceae*, associated with enhanced colonization rates and improved plant growth (Zhang et al., 2024). It was suggested that the altered exudate composition might provide an additional carbon source, favoring the recruitment of the microbial community at the rhizosphere (Zhang et al., 2024).

## 5 Host-specific effects on soil microbe recruitment and interactions

**Figure 3:**
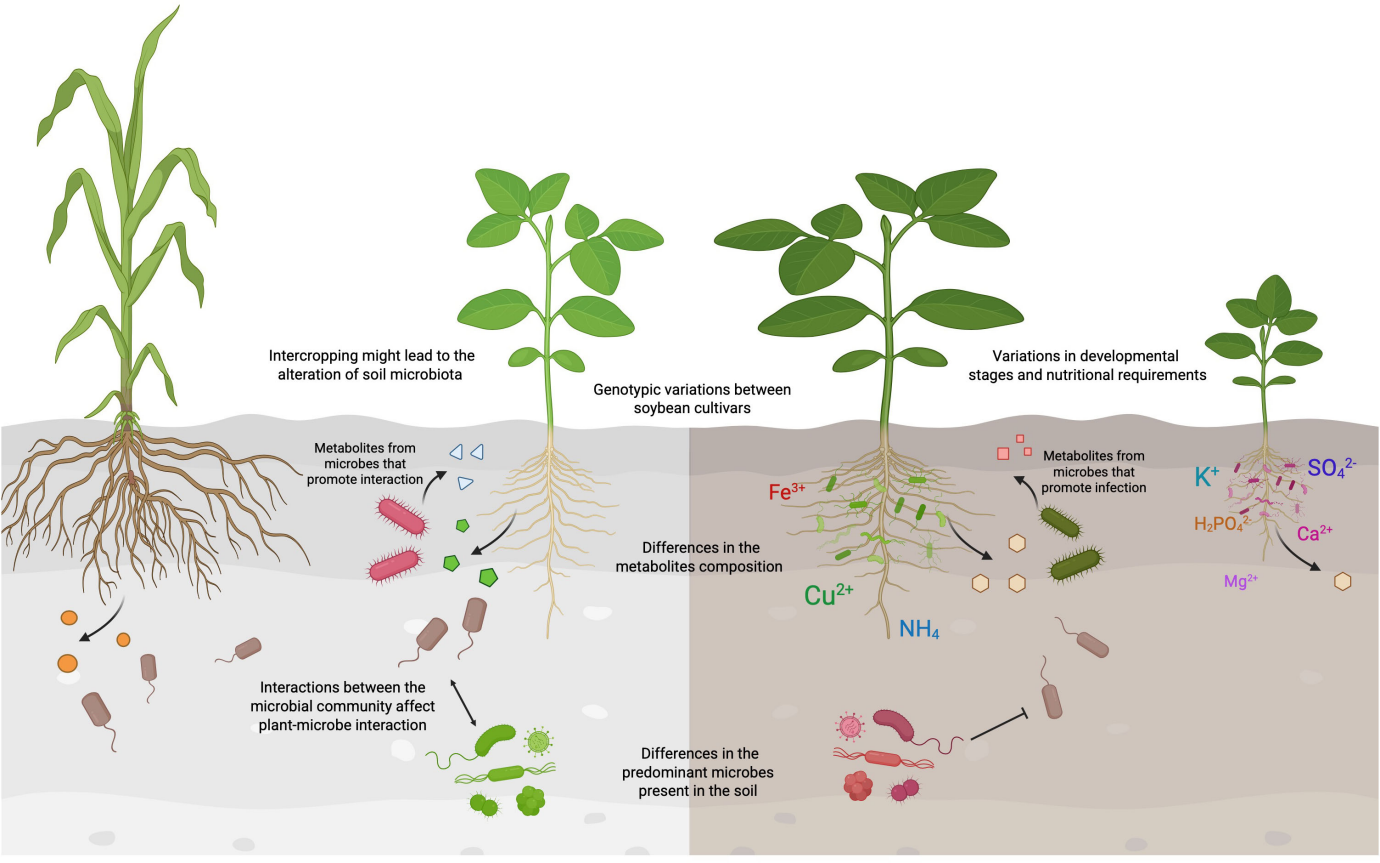
Factors affecting the interactions between soybean and soil microbes. Interactions between soybean plants and soil microbes can vary widely among different species due to many factors. Genotypic differences among soybean cultivars can lead to variations in the metabolites released into the rhizosphere, which may attract or deter distinct microbial populations. The soybean-interacting microbes might in turn secrete metabolites that modulate the gene expressions in soybean, either fostering beneficial interactions or causing infections. Soil properties, such as pH and the carbon-to-nitrogen ratio, play a crucial role in determining the types of microbes present in the soil. Furthermore, the agricultural and land-use history could also affect the microbial communities, thereby limiting the types of interactions that could occur between soybean and the soil microbiota. The interactions among microbes could also affect how soybean interacts with other microbial species. This figure was created using BioRender (https://BioRender.com/dttxnvt).

Genotypic variations might influence the specificity and efficiency of the interactions functional symbionts (Abulfaraj and Jalal, 2021; Oliveira et al., 2022; Begum et al., 2023). Therefore, interactions between species may not be universally successful, as shown in **Figure 3**. For example, the AMF *Rhizophagus clarus* inoculant preferentially colonized the soybean cultivar Desafio compared to Anta82 under drought conditions (Oliveira et al., 2022). It is proposed that different genotypes of the same plant species might exhibit altered gene expressions that affect their interactions with microbes (Begum et al., 2023). SQUAMOSA PROMOTER-BINDING PROTEIN-LIKE (SPL) proteins play a crucial role in improving the physiological development and architecture of plants (Chen et al., 2010; Sun et al., 2019). The *GmSPL9d*-expressing transgenic soybean demonstrated enhanced photosynthetic activities and growth parameters compared to their wild-type genetic background when inoculated with the AMF *R. irregularis* under drought conditions (Begum et al., 2023).

The outcome of the interactions between microbes and soybean plants can vary depending on the specific microbes and soybean genotypes involved. Inoculating soybean cultivars with *Pseudomonas fluorescens, P. putida*, or *B. subtilis* showed varying levels of effectiveness in ameliorating salinity stress (Abulfaraj and Jalal, 2021). Notably, *P. fluorescens* inoculation showed significant proline induction in soybean at 200 mM NaCl, whereas the proline level were induced by *P. putida* and *B. subtilis* only at 400 mM NaCl (Abulfaraj and Jalal, 2021). Furthermore, the inoculation of *P. fluorescens* led to higher catalase, ascorbate peroxidase and glutathione reductase activities in the salt-tolerant cultivar Crawford than in the less tolerant Giza-111 and Clark (Abulfaraj and Jalal, 2021). Endophytic *Bacillus licheniformis* P2.3 and the rhizobacterium *Bacillus aerius* S2.14 have been shown to positively interact with the soybean host, exhibiting various plant growth-promoting properties (Roriz et al., 2023). However, only the inoculation of *B. licheniformis* P2.3 increased pod numbers at maturity compared to the control under alkaline conditions (Roriz et al., 2023). Moreover, the inoculation of *B. licheniformis* P2.3 showed induced expression levels of iron nutrition-related genes, such as *IRT1, F6′H1, bHLH38*, and *FER4*, in soybean, facilitating iron solubilization and uptake under alkaline conditions (Roriz et al., 2023).

Different plant genotypes recruit distinct soil microorganisms to their rhizosphere, resulting in varied microbial community compositions in the rhizosphere (Wen et al., 2023; Yuan et al., 2023; Gouli et al., 2024; Zheng et al., 2024). Notably, the soybean cultivars AG49XF3 and P5554R were associated with unique endophytic bacterial communities, with similar patterns observed between the desiccated and surviving root tissues from these cultivars sampled from different locations under drought conditions (Gouli et al., 2024). The genera *Proteobacteria*, *Pseudomonas*, and *Pantoea* were predominantly enriched in the surviving root tissues, whilst *Streptomyces* was conspicuously dominant in the desiccated root tissues (Gouli et al., 2024). Research has shown that the decrease in soil enzyme activities and changes in the microbial structure of the soybean rhizosphere due to salt stress are influenced by different soybean genotypes (Han et al., 2023). Under salt stress, the salt-resistant soybean cultivar Qinong7, and the salt-sensitive soybean cultivar Hefeng50, recruited different microbial communities to their rhizosphere (Han et al., 2023; Yuan et al., 2023). The salt-resistant Qinong7 exhibited higher fungal abundances of *Talaromyces* and *Cladosporium*. Moreover, applying *Talaromyces* and *Cladosporium* to salt-sensitive Hefeng50 alleviated the symptoms of salt sensitivity (Yuan et al., 2023). Similarly, another study found that Qinong7 enriched a higher abundance of salt-resistant microbes, such as *Alicyclobacillus*, *Tumebacillus*, and *Bacillus*, than salt-sensitive Hefeng50, which might have contributed to enhanced salt tolerance in soybeans (Han et al., 2023). The continuous cropping-tolerant cultivars Qinong1 and Qinong5, and the continuous cropping-sensitive cultivars Heihe43 and Henong76 showed differences in the microbial compositions in their rhizospheres. The continuous cropping-tolerant cultivars showed reduced and increased relative abundances of *Acidobacteria* and *Proteobacteria*, respectively, under a 36-year continuous cropping system (Yuan et al., 2021).

Similarly, the aluminium (Al)-sensitive soybean cultivar BD2 exhibited a decrease in the pathogenic fungus *Nigrospora oryzae* and an increase in beneficial fungi such as *Sinomonas atrocyanea*, *Aquincola tertiaricarbonis*, and *Talaromyces verruculosus* (a phosphorus-solubilizing fungus) when inoculated with the AMF *Rhizophagus intraradices* and *Funneliformis mosseae* (Wen et al., 2023). On the other hand, the Al-tolerant soybean cultivar BX10 showed reduced microbial biodiversity in its rhizosphere upon the AMF inoculation. Nevertheless, four PGPRs were enriched, including *Chitinophagaceae bacterium* 4GSH07, *Paraburkholderia soli*, *Sinomonas atrocyanea*, and *Aquincola tertiaricarbonis* (Wen et al., 2023). Furthermore, higher levels of citric acid and malic acid were reported in the root exudate of the Al-tolerant cultivar BX10 than in the Al-sensitive cultivar BD2 (Yang et al., 2012), which chelate with the Al ions to reduce their availability and the potential aluminum toxicity to soybean (Rasheed et al., 2023). Transgenic soybean mutant overexpressing *GsMYB10* demonstrated enhanced Al tolerance by recruiting the beneficial members of *Bacillus*, *Aspergillus* and *Talaromyces* under Al toxicity (Liu et al., 2023). Application of the synthetic microbial communities composed of the enriched microbes in the *GsMYB*-overexpressing soybean leads to the upregulation of the genes involved in the organic acid transport in wild-type soybean with improved tolerance towards Al stress (Liu et al., 2023).Therefore, the root exudate composition from different soybean cultivars might contribute to the host-dependent effects on the microsymbionts (Yang et al., 2012; Wen et al., 2023).

AMF-plant interactions might upregulate the expressions of mitogen-activated protein kinases (MAPKs) and activate these pathways in the host cells. Several MAPK signaling components are encoded and expressed in the *Glomus intraradices* genome, suggesting their possible functional complementation or interference with the MAPK signaling pathway in plants (Tisserant et al., 2012; Liu et al., 2015). As an example, mycorrhizal soybean roots showed higher expressions of *GmMAPK2*, *GmMAPK3-2*, *GmMAPK4-2*, and *GmMAPK5* than in nonmycorrhizal soybean roots under well-watered conditions. Furthermore, AMF inoculation led to the upregulation of *GmMAPK2*, *GmMAPK3-2*, and *GmMAPK5* under drought stress (Liu et al., 2015). Meanwhile, expressions of *GiMAPK1* and *GiMAPK3* from the AMF were also induced under drought conditions. The expressions of the *GmMAPKs* and *GiMAPKs* appeared to be inversely associated, suggesting a potential interaction between the molecular pathways of MAPK signaling between the microbe and the host (Liu et al., 2015).

## 6 Conclusion

Soybean-microbe interactions are complex and dynamic, significantly influenced by the secretion of various compounds by both microbes and plants in the rhizosphere. Root exudates play a dual role of both facilitating and hindering such interactions. These exudates act as signals, mediating an underground communication network that connects neighboring plants with a wide range of microbes.

While extensive studies have focused on plant-to-microbe communication, there is still limited understanding on how beneficial microbes interact with one another, and associate with their plant hosts. Although many studies have explored specific metabolites and their roles in other plant models, research on soybean remains sparse.

Understanding the mechanisms by which microbes colonize and grow in the soybean rhizosphere, particularly under environmental stresses, will provide more insight into these fascinating but as-yet-poorly understood interactions between beneficial and pathogenic microbes. Filling this knowledge gap will not only enhance our comprehension of these ecological relationships but also lay the groundwork for developing sustainable agricultural practices. Leveraging these interactions can improve crop resilience and yield in an ever-changing environment.
